# The efectiveness of perioperative abdominal wall exercises upon functional recovery and return to work after Lichtenstein tension – free repair: a prospective randomized case –control study

**DOI:** 10.1007/s10029-026-03729-0

**Published:** 2026-06-02

**Authors:** R. Trisca, Valentin Oprea, M. Toma, I. Cadar, C. E. Bucuri, F. Finascu, O. Andercou, F. Bodog, I. R. Matei, C. Siserman, O. Chiroban, C. Gherman

**Affiliations:** 1 “Iuliu Hatieganu” University of Medicine and Pharmacy, Cluj- Napoca, Romania; 2 Clinical County Recovery Hospital, Cluj-Napoca, Romania; 3 “Constantin Papilian” Emergency Clinical Military Hospital, No22 Gral Traian Mosoiu Street, Cluj-Napoca, Romania; 4 University of Medicine and Pharmacy, Oradea, Romania

**Keywords:** Inguinal hernia, Lichtenstein repair, Convalescence, Physical rehabilitation, Return to work

## Abstract

**Background:**

Symptomatic groin hernias typically require surgical intervention, through either open or minimally invasive techniques. Among these, the Lichtenstein tension-free repair remains the gold standard for open procedures and is widely adopted today. Conventionally, patients are advised on convalescence and the timeline for resuming daily activities according with surgeons expertize. This study is based on the premise that structured physical rehabilitation can accelerate recovery and can facilitate an earlier return to work. Therefore, the goal was to evaluate the impact of a combined preoperative and post-operative exercise program as there is limited data that prehabilitation may decrease the period of convalescence.

**Materials and methods:**

A prospective randomized case –control study was conducted between January 1, 2023, and December 31, 2024, enrolling patients with primary unilateral reducible non-scrotal groin hernias. Participants were blindly randomised according to rehabilitation in a study group (receiving physical rehabilitation) and a control group (without rehabilitation). Baseline parameters including age, gender BMI (kg/m^2^), comorbidities and symptoms onset were recorded for all participants. Preoperative hernia - induced disability was assessed using the Pain Disability Index (PDI).Abdominal wall functionality was evaluated through clinical validated tests: Trunk Raising (TR), Double Leg Lowering (DLL), and a Total Score (TS) calculated as the sum of TR and DLL. The assessments were performed preoperatively, as well as at 7 and 30 days postoperatively. Acute postoperative pain was recorded at 24 and 72 h using the Visual Analogue Scale (VAS). The primary endpoint was the time to return to work, assessing the overall impact of rehabilitation on convalescence.

**Results:**

A total of 194 patients (97 per group) were analysed. Preoperatively, the study group exhibited significantly higher disability (PDI 56.7 ± 2.51 vs. 53.05 ± 2.74; *p* < 0.001) and lower abdominal functionality (AWF 5.15 ± 1.4 vs. 6.48 ± 1.34; *p* < 0.001) compared to controls, with baseline pain levels remaining similar (*p* = 0.937). Postoperatively, at 24 h, the control group experienced significantly higher VAS scores, particularly during mobilization (*p* < 0.001). By day 7, PDI reduction was markedly more pronounced in the rehabilitation group (18.48 ± 4.31 vs. 13.59 ± 3.83; *p* < 0.001), with a higher proportion of patients achieving the Minimal Important Change (61 vs. 42; *p* = 0.006). While AWF scores decreased significantly in controls by day 7 (*p* < 0.001), the study group maintained stability, reaching significantly higher mean AWF scores (7.77 ± 1.02 vs. 7.29 ± 0.92; *p* < 0.001) and superior functional recovery by day 30 (*p* = 0.01). Crucially, the rehabilitation group returned to work significantly earlier (9.28 ± 4.47 vs. 12.86 ± 5.16 days; *p* < 0.001). Multivariate regression identified preoperative PDI, functionality score, and symptom onset as independent predictors for both acute pain and early return to work (*p* < 0.05). During a mean follow-up of 18.4 ± 3.6 months, no recurrences were reported, and minor complications (hematoma, seroma, chronic pain) showed no significant differences between groups (*p* > 0.05), confirming the protocol’s safety.

**Conclusions:**

Rehabilitation with physical therapy reduce disability, increase abdominal function and reduces the convalescence period after groin hernia repair in accordance with the actual guidelines.

**Supplementary information:**

The online version contains supplementary material available at 10.1007/s10029-026-03729-0.

## Background

Inguinal hernia is one of the most common surgeries; almost 20 million repairs are performed each year over the world [1]. Despite advancements in surgical techniques, including minimally invasive surgery (MIS), the procedure remains associated with significant postoperative pain. The groin and abdominal wall constitute a fundamental muscle complex involved in nearly every human movement—from basic functions like breathing, sitting, and standing, to more demanding activities such as bending and walking. Surgical intervention inevitably causes tissue trauma to these muscle groups, requiring a complete healing process before pre-operative functional capacity can be fully restored. Consequently, the core of postoperative management often evolves around three critical patient concerns: When can I resume physical activity? What weight am I allowed lifting? and When can I return to work? [2, 3]. Addressing these questions is essential for an optimal patient satisfaction.

Current clinical recommendations remain highly heterogeneous, inconsistent, and often lack a solid evidence-based foundation, frequently leaning toward excessive restrictiveness. The critical issue of physical activity restriction remains significantly under-researched and the appropriate duration of convalescence has long been poorly understood and a subject of controversy [4]. Historically, recommendations have relied on retrospective, observational studies and are deeply rooted in clinical tradition rather than data [5, 6]. Currently, there is no robust evidence to justify overly restrictive postoperative protocols. Such counselling is typically driven by common clinical apprehensions, specifically the fear of recurrence, potential mesh-related complications, and the management of postoperative pain. Current clinical guidelines emphasize that patients should be encouraged to resume their baseline physical activity levels as soon as possible, guided primarily by their pain tolerance but the statement is not evidence based [7].

The goal of our study was to assess prospectively the effectiveness of preoperative and postoperative optimization of physical condition through targeted exercises on postoperative pain, return to work and daily activities in patients with a Lichtenstein tension free repair. We hypothesize that preoperative conditioning with a structured rehabilitation program, facilitates faster recovery, an earlier return to activity, and reduces pain level. This was evaluated by measuring the performance of the muscle groups most affected by inguinal hernia surgery utilising specific exercise tests and clinical evaluation of the abdominal wall functionality.

## Patients and methods

### Study design and population

This was a prospective randomized case – control observational study conducted between January 1, 2023, and December 31, 2024involving patients undergoing elective repair for primary, unilateral, and reducible non-scrotal inguinal hernias. The study included patients aged 18 years and older. Paediatric patients, patients with a history of psychiatric disorders or cognitive impairments, those with restricted mobility or recurrent hernias and complicated emergency cases were excluded. Additionally, patients unable to complete the prescribed physical program were excluded for the final analysis (personal reasons, too difficult to perform, no available time). Patients were randomly assigned to one of the two groups: **group A (RP – study group)** which underwent the structured rehabilitation protocol and **group B (WR - control)** which received no rehabilitation. Randomization was computer - generated using the Research Randomizer software (https://www.randomizer.org/). To ensure concealment, the allocation results were placed in sequentially numbered, opaque, sealed envelopes. After signing the informed consent and agreeing to participate, each patient received an envelope; it was opened only at that point to determine their assignment to either the rehabilitation or the control group. Randomization was implemented to ensure an unbiased distribution of participants and to achieve baseline comparability between the two groups. Any observed improvements in recovery time or pain reduction can be statistically attributed to the physical exercise protocol rather than to the natural healing process or placebo effect.

The study was conducted in accordance with the guidelines of the Declaration of Helsinki, and the local Ethics Committee approved the study protocol (reference number: A2163/November 2022). All participants gave written informed consent before being included in the study. An independent reviewer (FF) who was unaware which group the patients were assigned to, performed the postoperative follow-up in the 7th, and 30th days. The operating surgeon clinically examined the patients at 6 and 12 months.

### Sample size

The sample size was calculated based on a two-tailed comparison of proportions between the two study groups, using a 1:1 ratio [8]. The observed incidence of postoperative pain was 52%, and an expected reduction of 60% was anticipated with the abdominal wall strengthening exercise. With a significance level (alpha) of 5%, a power (beta) of 80%, and an estimated dropout rate of approximately 10%, the calculated sample size was 93 patients per group.

### Data collection

Study variables encompassed *patient-related factors*, including age, gender, Body Mass Index (BMI), occupational status, and the onset of symptoms. Relevant comorbidities, such as arterial hypertension, diabetes mellitus, and smoking history, were also documented. *Intraoperative parameters* recorded included hernia location, the width of the fascial defect, the content of the hernia sac (where applicable), and the total duration of the surgical procedure. *Clinical outcomes* were evaluated using the Pain Disability Index (PDI), which was assessed preoperatively and at multiple postoperative intervals: 7 days, 30 days, and 12 months.

### Description of physical rehabilitation

The rehabilitation protocol was specifically designed to provide maximum therapeutic benefit while ensuring patient safety and ease of implementation. The program consisted of six targeted exercises initiated four weeks prior to surgery. To ensure adherence and mitigate any potential risks, the program was supervised by an experienced physical therapist (I.C.), who monitored each patient’s progress. The exercise intensity was increased progressively: Week 1: 3 repetitions per exercise; Week 2: 5 repetitions; Week 3: 7 repetitions; Week 4: 10 repetitions. Patients received a one-hour introductory session and demonstration to ensure proper technique and task comprehension. On the third postoperative day, the protocol was resumed following the same incremental structure. Rehabilitation was conducted under professional supervision during the first postoperative week and continued independently at home thereafter, mirroring the preoperative phase.

**Fig. 1:**
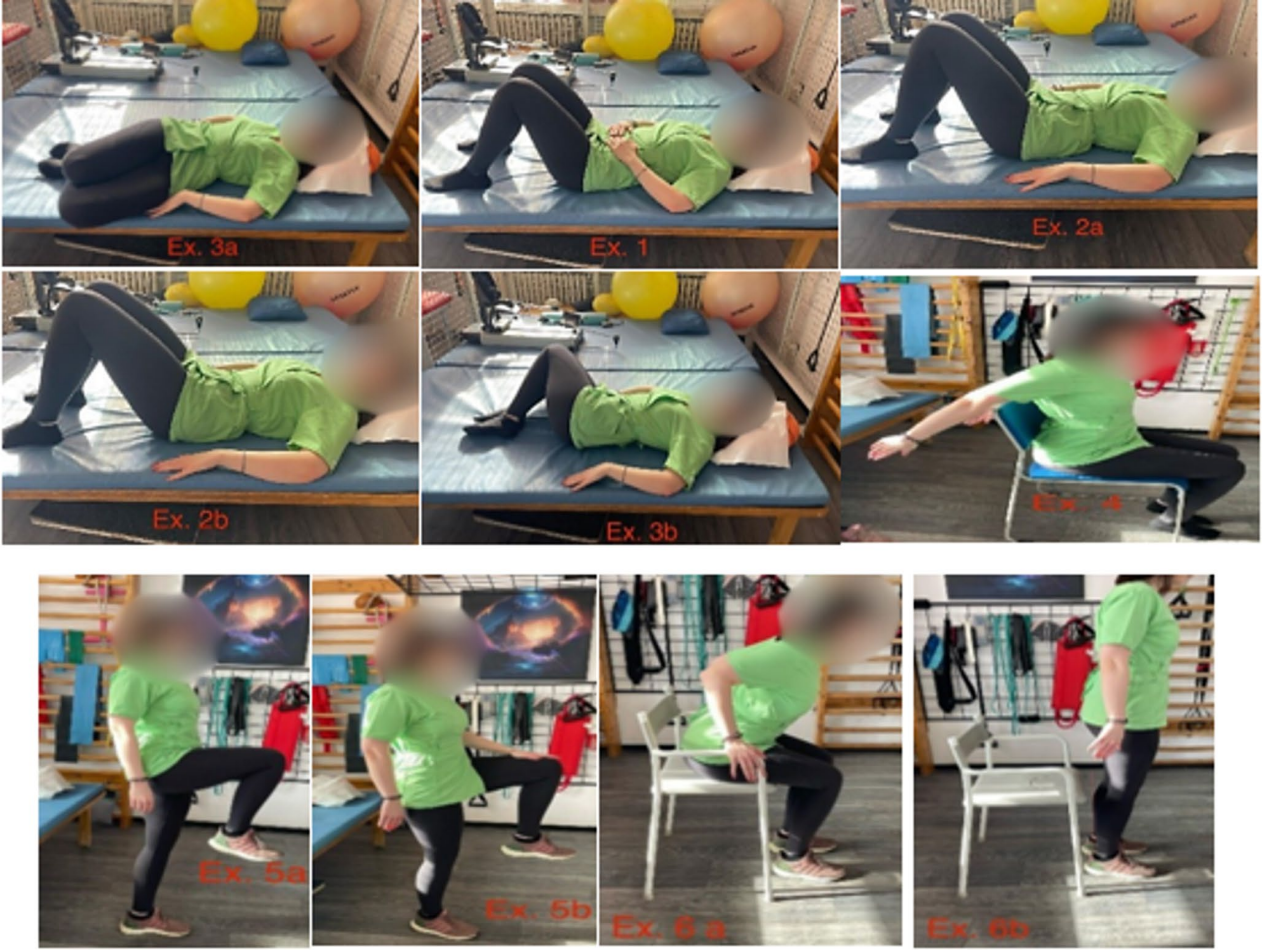
A brief demonstration of the program of physical exercises

The physical program included:

1. ***Connect with the trunk***: In supine position with flexed legs and hands on the abdomen, the patient breathes very slowly, lengthening the exhalation until feel a slight contraction of the deep abdominal musculature. This contraction should last 3 to 5 seconds before exhaling. This exercise works external, internal, and transverse muscles of the abdomen.
2. ***Pelvic Rotation***: In supine position with flexed legs, the patient performs pelvic retroversion and ante version while keeping the legs relaxed. This exercise works rectus abdominis muscle.
3. ***Knee rotation***: In supine position with flexed legs, the patient performs a lateral inclination of the knees with the legs bent. This exercise works internal and external oblique muscles working unilaterally.
4. ***Elevation of the arm***: Sitting, with the back as straight as possible, the patient slowly raises and lowers the arm in full extension. This exercise works rectus femoris muscle.
5. ***Elevation of knees***: Sitting, in upright posture, the patient slowly flexes the hip with the knee flexed. Initially, the exercise is done without resistance and then applying progressive resistance by the patient with his hand. This exercise works psoas-iliac muscle.
6. ***Get up from the chair***: Sitting on the edge of the chair (or bed, at first) with hands on the armrest (if available) or on the thighs, the patient performs a slight inclination of the trunk forward, looking up. Then, using this inclination as an impulse, stands up, placing one foot slightly forward. This exercise works quadriceps, gluteus maximus and trunk erectors muscles. A brief description of the physical program can be found in Fig. 1.

### Abdominal wall functionality (AWF)

Abdominal wall functionality (AWF) was assessed using two validated clinical tests: Trunk Raising (TR) and Double Leg Lowering (DLL), as previously described in the literature [9]. Given the proven reliability of these tests, we extended their application to groin hernia repairs. To ensure consistency and inter-ratter reliability, all measurements were performed by a dedicated team of two examiners (OV and MT), both of whom conducted the assessments for every participant. The AWF value was calculated as a composite score, representing the sum of the TR and DLL results (detailed in Table 1). Data collection was performed at four specific intervals: **preoperatively**, and postoperatively at 7 days, 30 days, and 1 year.

**Table 1:** Abdominal wall functionality combined score evaluation (the combined score is the sum of the value of trunk raising (TR) and double leg lowering (DLL)

TRACE	1–2 points
POOR	3–4 points
FAIR	5–6 points
GOOD	7–8 points
NORMAL	9–10 points

### Disability evaluation

Subjective pain-related disability was assessed using the Pain Disability Index (PDI). The PDI is a validated 7-item questionnaire designed to measure the magnitude of self-reported, pain-related interference with daily activities, independent of the pain’s anatomical region or specific diagnosis. Each of the seven items is scored on a 0–10 numeric rating scale, where 0 represents no disability and 10 signifies maximum disability. The total PDI score is calculated as the sum of these items, ranging from 0 to 70, with higher scores reflecting a greater impact of pain on functional capacity. Each of the seven items is scored on a 0–10 numeric rating scale, where 0 represents no disability and 10 signifies maximum disability. The total PDI score is calculated as the sum of these items, ranging from 0 to 70, with higher scores reflecting a greater impact of pain on functional capacity. The PDI evaluates disability across seven distinct life domains: 1.Family/Home responsibilities; 2.Recreation; 3.Social activity; 4.Occupation; 5.Sexual behavior; 6.Self-care, and 7.Life-support activities [10, 11]. Here is a more detailed breakdown of PDI score interpretation:

- **0-10:** *Minimal* disability. Pain has little impact on daily activities.
- **11-20:** *Mild* disability. Pain causes some limitations in daily activities.
- **21-30:** *Moderate* disability. Pain significantly affects daily life, causing noticeable limitations.
- **31-40:** *Moderately severe* disability. Pain significantly disturbs many aspects of daily life.
- **41-50:** *Severe disability*. Pain causes substantial limitations in daily activities and may require assistance.
- **51-60:** *Very severe disability*. Pain severely restricts daily activities and significantly impacts quality of life.
- **61-70:** *Extremely severe *disability. Pain is debilitating and severely limits almost all activities.

Missing items were resolved as follows: patients were allowed to miss no more than one question on the PDI. In this case, the missing value was replaced by the patient cluster mean. As the PDI only consists of seven questions, the patient was excluded from the study if more than one question on the questionnaire was missed.

The test was translated for our population and applied to 28 patients with inguinal hernias for internal validation in 2020. Cronbach’s α was 0.78 suggesting a shared dimensions of the items. For the test – retest reliability, participants completed the questionnaires after 3.9 ± 0.97 h (mean ± SD) apart. The ICC was found to be 0.88 (95% CI: 0.79–0.89). Based on these data we considered that the test could meet our pain evaluation needs with minimal risk of bias.

Additionally, pain (at rest and after mobilization) was independently evaluated before surgery and 24, 72 h after surgery, at 7 and 30 days with the aid of a 10 cm length VAS scale. Pain was evaluated as **no pain** (0–1 cm), **mild pain** (2–4 cm), **moderate** (5–7) and **severe** (8–10).

### Surgical procedure

All the patients were admitted the night before surgery and a dose of 0,4 ml of Low Molecular Weight Heparin (Fraxiparine™ - Aspen Pharma) was administered. No antibiotic prophylaxis was administered before surgery. All procedures were performed under spinal anaesthesia for all patients by a single surgeon (OV). A variable length incision (6 to 10 cm. according to hernia volume) was performed as bisector of the angle created by lateral rectus muscle margin and the line between anterior iliac superior spine and pubis. The inguinal canal was opened by transection of the external oblique aponeurosis. All the inguinal nerves were identified and preserved. If the mesh deployment was embarrassed by the nerve’s location, they were cut on their entire inguinal length, ligated with a 3 − 0 absorbable Polyglycolic suture and embedded in the internal oblique muscle. The cord enveloped in the cremasteric fascia, was bluntly dissected from the posterior wall with a mounted sponge and lifted on a rubber band with the preservation of the genital branch and of the cremasteric vessels (the “blue line”). For indirect hernias, the sac was opened and the content reduced in the peritoneal cavity. Sac dissection was performed in the proximity of the deep ring (DR) and deep in the preperitoneal space. After that, it was transected and the proximal stump was ligated with an absorbable suture. The remnant distal sac was abandoned after careful haemostasis. In sliding hernias, the sac was fully dissected and inverted. The direct hernia sac was inverted in a “purse – string” suture. Preperitoneal space was routinely explored in all patients through the DR to identify a femoral hernia. The groin defect was classified according with the sac location as indirect (L: L1 < 1.5 cm, L2 = 1.5–3 cm and L3 > 3 cm), direct (M: M1 < 1.5 cm, M2 = 1.5–3 cm and M3 > 3 cm), femoral (F: F1 < 1.5 cm, F2 = 1.5–3 cm and F3 > 3 cm) and its dimensions in the largest diameter were measured. Groin defect was corrected according to Lichtenstein repair as described by Amid et al.l [12]. A flat sheet of macro porous monofilament polypropylene standard medium weight (45 g/m^2^ ) was used (Dipromed Evolution – Turin, Italy). A flat peace of mesh (13 × 7 cm) was cut along its lateral edge to create two unequal “tails”: a lower one narrower (1/3 of the width) and an upper one, closest to the internal oblique, wider (2/3 of the width). That will reconstitute the mesh internal ring around the cord [12]. and it was fixed with synthetic cyanoacrylate glue (Hystoacril ™ B Braun Melsungen) or with 2 − 0 non – absorbable monofilament polypropylene sutures (Monoplus™ B Braun) according with surgeons’ choice. A non-absorbable 2 − 0 monofilament suture was used, starting 1 cm caudal to the symphysis pubis and run in continuous fashion to the level of the internal ring to fixate the lateral edge of the mesh to the inguinal ligament. For securing mesh to the conjoint tendon/transversus arch a resorbable 2 − 0 suture (Polyglycolic acid) was used [12]. Glue fixation was performed in the same manner. No drains are used.

Perioperative pain relief Tenoxicam 40 mg was given orally the evening before and two hours preoperatively followed by 20 mg daily for the following four days.

### Outcomes of interest

Date to return at normal activity (in days after procedure) and quality of life expressed as PDI were the main outcomes of interest. Secondary outcomes were 30-day wound events and general complications including 90 days mortality. Postoperative wound events included Surgical Site Infection (SSI), Surgical Site Occurrence (SSO), and Surgical Site Occurrences Requiring Procedural Intervention (SSOPI). According to the Centre for Disease Control and Prevention (CDC), SSI was defined as superficial, deep or organ space [13]. Surgical Site Occurrence included any SSI, in addition to wound cellulitis, non-healing surgical wound, skin or tissue ischemia, skin or soft tissue necrosis, fascial disruption, serous or purulent wound drainage, suture abscess, seroma, hematoma, infected or exposed mesh, enterocutaneous fistula. Procedural interventions which were considered SSOPI included wound opening and/or debridement, stich removal, percutaneous drainage, partial and/or complete mesh removal. Length of hospital stay, 30-day readmission and mortality were also analysed. Patients having multiple wound complications such seroma and infection, dehiscence and infection, were classified as the most severe. The patients were examined in the outpatient clinic at 6 months, 12 months, and yearly or whenever necessary if clinical complaints were described. A recurrence was defined as a bulge at the operative site at any moment of the follow-up; clinical suspicion was confirmed by an ultrasound exam or a native CT abdominal – pelvic scan. All postoperative data were collected unblinded.

### Statistical analysis

Data were tabulated as mean ± standard deviation (SD). Continuous variables were analysed by ANOVA variance test followed by unpaired two tails Student’s t test assuming unequal variance and the binary outcomes with the Chi-square (χ2) test. Pearson correlation coefficients with the regression equation were used to estimate the correlation between different cofounders. Correlations were considered ‘strong’ if the coefficient value lied between 0.50 and 1, were said to be ‘moderate’ if the value lied between 0.30 and 0.49; and were considered ‘weak’ correlations if the coefficient lied below 0.29. Multivariate logistic regression models were built for outcomes of interest adjusting for identified confounders if a significant correlation was found in univariate analysis. In addition to the variables of interest, the following were included for adjustment: age, severity of comorbidities score, BMI, width of the defect, and pain. Multivariate models were calculated with the linear logistic regression and the results were shown with the Odd’s ratio (OR) and the 95% confidence interval (CI). A Receiver Operating Characteristics (ROC) curve was designed to determine the specificity and sensitivity of the cofounders in relation with the total amount of AWF [14]. Youden index was determined to calculate the optimal cut-off value for each cofounder. Minimally important change (MIC) for PDI was calculated with the aid of ROC curve and Youden Index (J_max_) (15). Probabilities smaller than 0.05 were considered as statistically significant. SPSS statistic version 23.0, 2018 (Ch., Ill) was used to perform the statistical analysis.

## Results

**Fig. 2:**
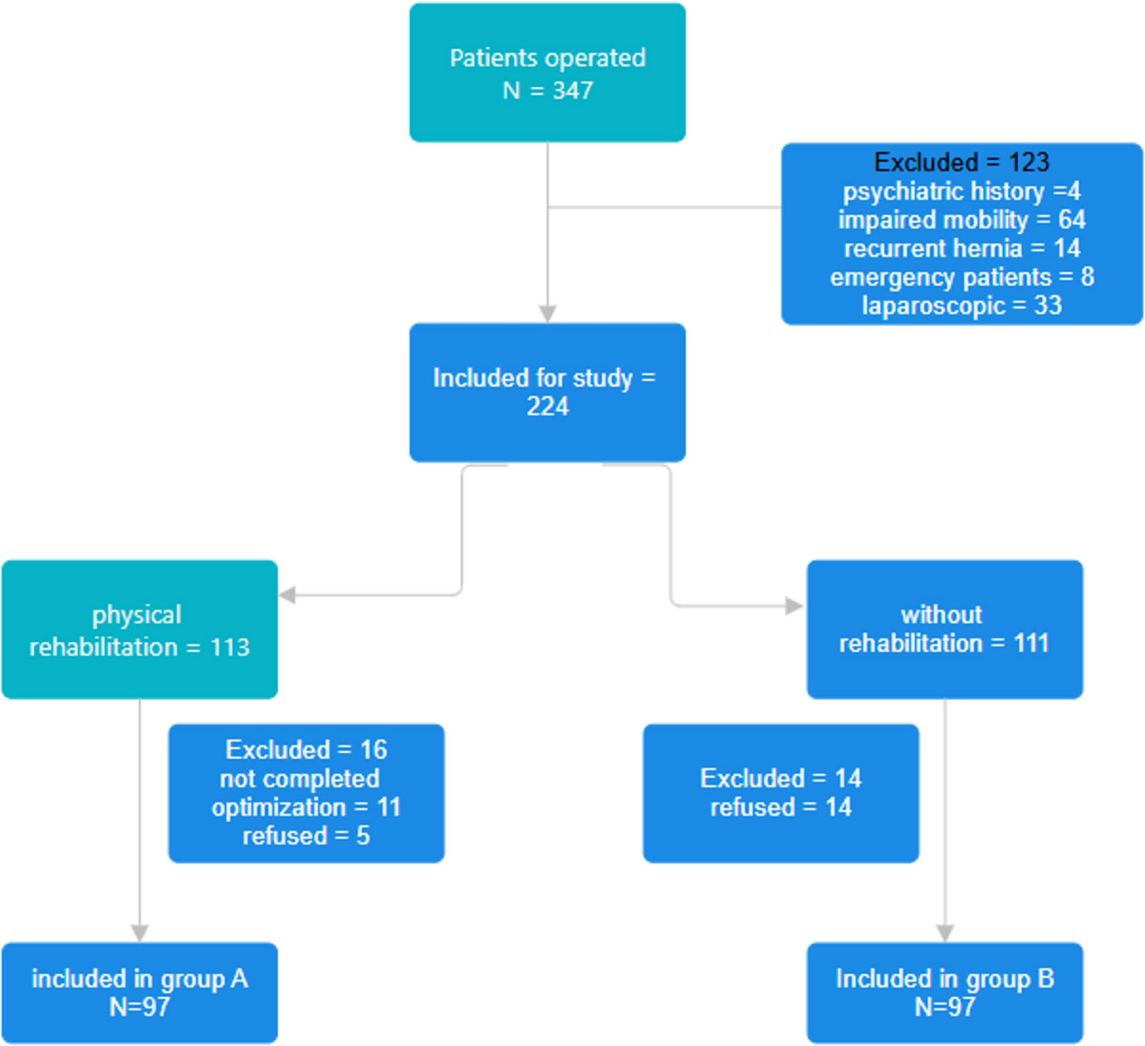
Flow chart of inclusion and exclusion criteria

In the referral interval, 347 patients where admitted and operated in the Department of Surgery. Of them, 194 agreed to participate, signed the informed content and were included in the study (Fig. 2). The mean age was 57.12 ± 9.95 years (23–82 years), and as expected, most of the patients were males (183–94.32%). Body Mass Index ranged between 17 and 44 Kg/m^2^ (mean 30.81 ± 5.22); there were 73 patients with a BMI between 30 and 35 Kg/m^2^ and 25 over 35 Kg/m^2^. According to the type of work, 49 performed light/medium work, 95 heavy work, and 50 were retired. Onset of symptoms varied between 3 and 309 month with a mean of 64.44 ± 61.68 months. 16% had undergone a prior repair of a contralateral hernia. After randomization, 97 patients were equally distributed for each group. Except the decreased number of retired patients of the study group, the distribution between groups in terms of demographics, comorbidities and hernia characteristics was homogenous (Table 2).

**Table 2:** Demographics of the included in the study. Data are eexpressed as means and standard deviation (SD). M/F – Male/Female; BMI – Body Mass Index; CVD – Cardiovascular Disease; COPD – Chronic Obstructive Pulmonary Disease

Variable	Prehabilitation group (study) *N* = 97	Without rehabilitation (control) *N*=97	Significance
Age (years) (mean SD)	59.96 ± 11.8	61.27±7.17	*p* =0.411
M/F ratio	90/7	93/4	*p* = 0.534
BMI (Kg/m^2^) (mean SD)	30.6±4.6 (21- 44)	31.0±5.7 (17 – 39)	*p* = 0.922
Onset of symptoms (months)	62.7±61.4 (3-282)	62.1±62.1 (4-309)	*p* = 0.279
Easy/medium work	29	20	*P* = 0.186
Heavy work	51	44	*P* = 0.388
Retired	17	33	*P* = 0.013
Comorbidities	Diabetes - 12 CVD – 19 COPD -7	Diabetes – 9 CVD – 13 COPD - 5	*p* = 0.311 *p*=0.197 *p*=0.363
ASA score	2 – 91 3 – 6	2 – 93 3 – 4	*P*=0.891
Hernia location			
Indirect			
L1(<1.5 cm)	19	14	*p* = 0.44
L2 (1.5–3 cm)	34	37	*p* = 0.765
L3 (>3 cm)	11	12	*p* = 1
Direct			
M1 (<1.5 cm)	4	4	*p* = 1
M2 (1.5–3 cm)	14	12	*p* = 0.83
M3 (>3 cm)	15	18	*p* = 0.702

### Disability (PDI)

Preoperatively, the study group presented higher disability (PDI 56.7 ± 2.51 vs. 53.05 ± 2.74; *p* < 0.001) and lower abdominal functionality (5.15 ± 1.4 vs. 6.48 ± 1.34; *p* < 0.001) than controls. Postoperatively, the study group achieved a significantly greater PDI reduction at 7 days (18.48 ± 4.31 vs. 13.59 ± 3.83; *p* < 0.001) and a higher proportion of normal functional recovery by day 30 (87 vs. 68 patients; *p* < 0.001). Multivariate regression identified preoperative PDI (OR = 0.03, 95%CI = 0.002–0.07; *p* = 0.002), functionality score (OR = 0.004, *p* < 0.001), and symptom onset (OR = 0.041, *p* = 0.001) as independent predictors of early return to work (Table 3). Minimally Important Change was positive if greater than 13 points for the study group while for the controls was 17 (*p* < 0.01). More details in supplemental material.

**Table 3:** The evolution of the PDI in different moments of evolution. The decrease was significant comparing to baseline (preoperative PDI) but a major improve was recorded in patients with prehabilitation. PDI – Pain Disability Index. Data are expressed as mean and standard deviation (SD). In brackets minimum and maximum recorded values

	Preoperative PDI	7 days PDI	30 days PDI	*p*
Study group *N* = 97	56.7 ± 2.51 (47–63)	38.34 ± 4.02 (29–52)	33.08 ± 4.97 (22–52)	**< 0.001**
Control group *N* = 97	53.05 ± 2.74 (43–59)	39.47 ± 3.25 (32–53)	34.14 ± 4.32 (23–49)	**< 0.001**
*p*	**< 0.001**	**0.004**	0.082	

### Pain

Preoperative baseline pain was minimal and similar between groups (*p* = 0.937). Postoperatively, the control group experienced significantly higher VAS scores at 24 h, particularly during mobilization, where 59 patients reported severe pain compared to only 20 in the study group (*p* < 0.001) (Table 4). Multivariate analysis identified preoperative PDI (OR = 0.078, *p* = 0.038), symptom onset (OR = 0.074, *p* = 0.027), and hernia defects > 3 cm (OR = 0.059, *p* = 0.003) as independent predictors of acute postoperative pain, demonstrating high sensitivity and specificity (AUROC = 0.762; *p* < 0.01).

**Table 4:** The evolution of the acute postoperative pain at rest and movement. The mean value was significantly lower for patients with rehabilitation. Data expressed as mean and standard deviation (SD). VAS – Visual Analogic Scale

	VAS 24 hours (rest)	VAS 24 hours(movement)	VAS 72 hours (rest)	VAS 72 hours (movement)
Study group *N* = 97	2.67±1.23(0 – 6)	4.46±1.56(2 – 7)	1.29±0.58(0 – 4)	2.23±0.47(1 – 4)
Control group *N*=97	3.85±1.52(0 – 7)	5.83±1.52(3 – 8)	4.15±1.14(2 – 7)	4.28±1.32(2 – 7)
	***P*<0.0001**	***P*<0.0001**	***P*<0.0001**	***P*<0.0001**

### Abdominal wall functionality

Baseline abdominal wall functionality (AWF) was predominantly fair or poor, independently predicted by preoperative PDI (*p* = 0.021) and symptom onset (*p* = 0.034). Postoperatively, AWF scores remained stable in the study group at 7 days but decreased significantly in controls (*p* < 0.001), correlating strongly with 72-hour mobilization pain (*p* = 0.05). By day 30, the rehabilitation group achieved significantly higher mean AWF scores (7.77 ± 1.02 vs. 7.29 ± 0.92; *p* < 0.001) and a superior rate of normal functional recovery (22 vs. nine patients; *p* = 0.01). Preoperative PDI was identified as the primary independent predictor of functional outcomes throughout the early postoperative period (*p* = 0.0012) (Table 5).

**Table 5:** The evolution of the abdominal wall functionality in baseline evaluation. Note the increased number of patients with “good function” in control group. Data expressed as mean and standard deviation (SD)

Variable	Study group *N* = 97	Control group *N* = 97	Significance
Trunk raising (TR)	2.42 ± 0.76 (1–4)	3.27 ± 0.85 (1–5)	
Double leg lowering (DLL)	2.73 ± 0.88 (1–4)	3.24 ± 0.85 (1–5)	
Total score (TS)	5.15 ± 1.4 (2–8)	6.48 ± 1.34 (3–10)	***P* < 0.0001**
Trace (1–2)	5	0	χ² = **5.13,** ***p*** = **0.023**
Poor (3–4)	24	5	χ² = **14.64,** ***p*** **< 0.001**
Fair (5–6)	52	44	χ² = 1.32, *p* = 0.251
Good (7–8)	16	43	χ² = **17.76,** ***p**** < ***0.001**
Normal (9–10)	0	5	χ² = **5.13,** ***p*= 0.023**

**Fig. 3:**
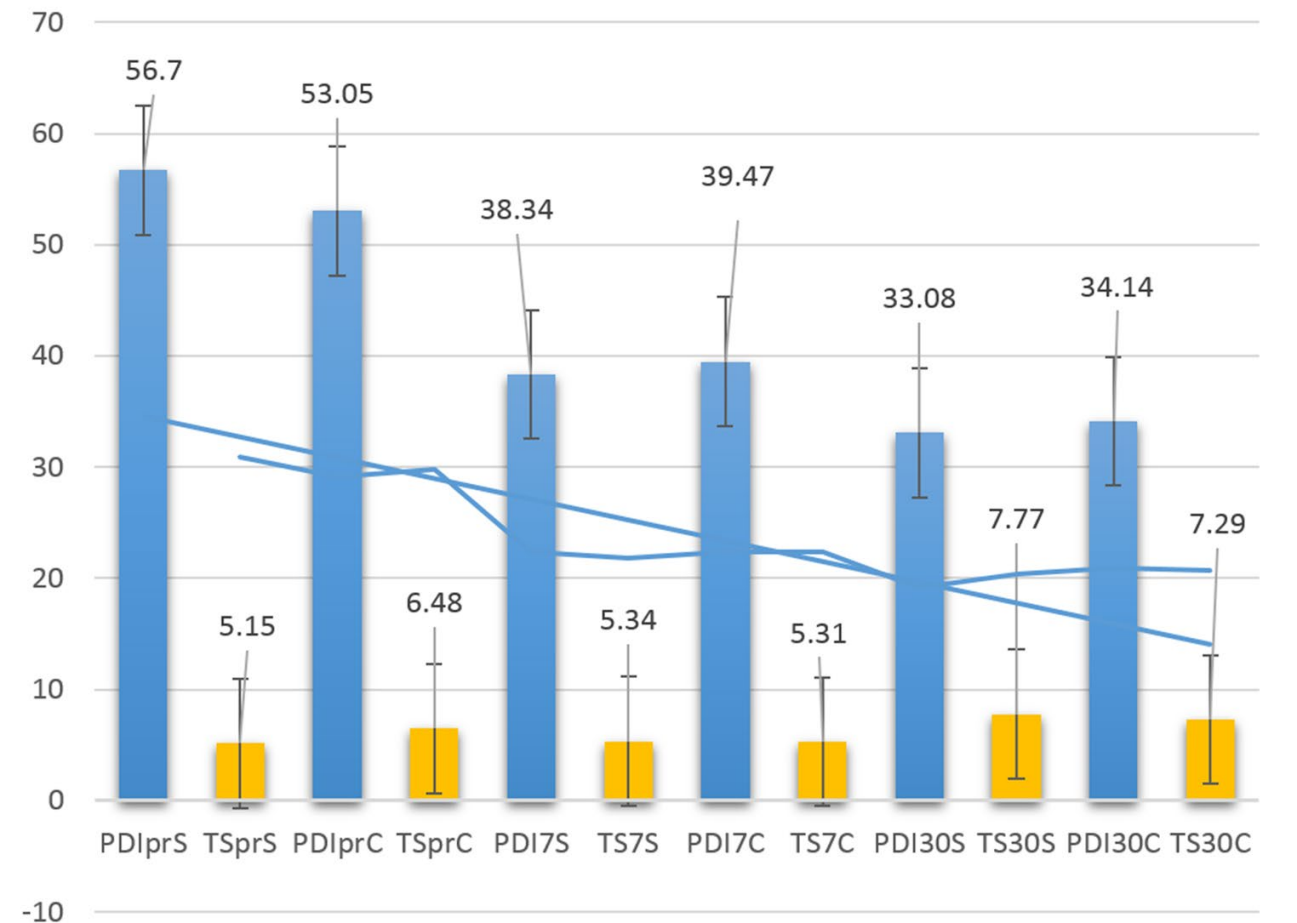
The relation between the decrease of PDI and increase of the total score of functionality during the evaluation period. PDI – Pain Disability Index; TS – Total Score of functionality. PrS/C – preoperative evaluation study/control; 7 S/C – 7-day evaluation study/control; 30 S/C – 30-day evaluation study/control

The relation between the values of PDI and TS is described in Fig. 3.

### Return to work

The rehabilitation group returned to work significantly earlier than controls (9.28 ± 4.47 vs. 12.86 ± 5.16 days; *p* < 0.001), with 49% of study patients resuming activities within 7 days compared to 22% of controls (*p* < 0.001) (Table 6). Multivariate logistic regression identified preoperative PDI (OR = 0.03, *p* = 0.002), functionality score (OR = 0.004, *p* < 0.001), and symptom onset (OR = 0.041, *p* = 0.001) as independent predictors of early return, explaining 61% of the variance. Specifically, an increase of 10 PDI points over the MIC was associated with two additional recovery days for heavy workers in the study group versus four days in the control group (*p* = 0.005). Similarly, a two-point decrease in functionality scores delayed return by two days in the rehabilitation group compared to five days in the control group (*p* = 0.004).

**Table 6:** The return to work of the patients according to the type of activity. More patients from the study group return earlier to work (less than 7 days) compared with the controls (χ^2^ = 8.756; *p* = 0.00271)

	Easy work		Heavy work		Retired	
	S	C	S	C	S	C
Under 7 days	19	6	19	7	11	8
Over 7 days	10	14	32	37	6	25
Total	**29**	**20**	**51**	**44**	**17**	**33**

### Follow-up and outcomes

During a mean follow-up of 18.4 ± 3.6 months, no clinical recurrences or acute hernia-related accidents were reported in either group. Postoperative complications were minor, including hematoma (*n* = 3), seroma (*n* = 9), wound dehiscence (*n* = 1), and chronic pain (*n* = 2), with no statistically significant differences in local events between the groups (*p* > 0.05). None of the reported complications required surgical intervention, confirming the safety of the rehabilitation protocol.

## Discussions

Inguinal hernia repair is one of the most frequently performed surgical procedures globally, with nearly 20 million repairs annually [15, 16]. Despite the evolution of tension-free techniques and laparoscopic approaches, which have significantly reduced length of hospital stay, recurrence rates and accelerated recovery, postoperative management remains inconsistent [17–19]. Traditionally, patients are advised to restrict physical activity for 6–8 weeks based on expert opinion rather than empirical evidence [4, 5, 20]. There is a lack of literature concerning return to work and convalescence after hernia repair and reliable, evidence based recommendations are almost absent [20]. This has led to a wide variation in expected time off work and restriction of normal activity [21]. Our study challenges such restrictive protocols, aligning with HerniaSurge Guidelines, which advocate for an immediate, pain-guided return to normal activities [7].

In 2004 a larger prospective, multicenter, nonrandomized study of over 1000 patients sought to investigate the consequences of a surgeon-recommended 1-day convalescence on recurrence and return-to-work rates. The median time off work in this study was 7 days (extended to 14 days for patients in the most strenuous occupations), with no increase in recurrence [22]. Of patients who had not returned to work by postoperative day 7, 64% cited pain and 17% cited wound complication as the reason. In a randomized trial published in the *Lancet*, the Medical Research Council of Great Britain noted a 37% incidence rate for residual pain after open repair compared with a 27% incidence rate following laparoscopic hernia repair [23].

Our findings demonstrate that structured pre- and postoperative rehabilitation significantly reduces the convalescence period. The rehabilitation group returned to work on average in 9.28 days, compared to 12.86 days in the control group (*p* < 0.001), values comparable and in accordance with HerniaSurge recommendations (7,26–30) and to those reported by Kark and Amid [7, 24–31]. A novel aspect of this research is the use of the Pain Disability Index (PDI) and abdominal wall functionality (AWF) as primary predictors of recovery. Our data indicate that preoperative disability levels and fascial defect size (> 3 cm) are independent factors influencing postoperative pain and return to work. By utilizing Patient-Reported Outcome Measures (PROMs), we shifted the assessment of surgical success from clinician-centered metrics to functional quality of life [32]. The PDI proved to be a reliable and responsive tool for evaluating hernia-induced disability for the first time in this surgical context [10, 11].

A core strength of this study is the application of the Minimal Important Change (MIC) to evaluate the real-world impact of both surgery and rehabilitation on the Pain Disability Index (PDI). By establishing a threshold for minimal within-person change, the MIC identifies the true number of ‘responders’ to the treatment, providing a meaningful interpretation of success from the patient’s perspective. In our cohort, over 50% of patients achieved an improvement exceeding the MIC, demonstrating the clinical validity of the rehabilitation protocol [33]. This patient-centred metric reinforces the shift toward Patient-Reported Outcome Measures (PROMs), moving beyond objective surgical success to focus on perceived functional recovery.

The implementation of this physical rehabilitation regimen requires a multidisciplinary approach, integrating a brief preoperative educational session (approx. one hour) where patients are taught the six-exercise protocol by a physical therapist [30]. To ensure scalability, the program can be delivered via digital platforms or instructional leaflets, allowing for home-based performance with minimal professional supervision, which has been shown to maintain high adherence rates in elective surgery [31]. While all patients undergoing Lichtenstein repair benefit from early mobilization, our data suggest that heavy manual workers and those with high baseline disability (PDI > 50) derive the most significant clinical gain, showing a more pronounced reduction in convalescence compared to sedentary individuals [29]. Furthermore, patients with large fascial defects (> 3 cm) or **a** prolonged onset of symptoms should be prioritized for this intervention, as these factors were independent predictors of delayed recovery and increased postoperative pain. Adopting this ‘prehabilitation’ model facilitates a shift from traditional, restrictive advice toward an evidence-based, pain-guided recovery, ultimately reducing the global socio-economic burden of hernia-related absenteeism [7, 19].

### Strengths of the study

The use of a randomized case-control design with concealed allocation (sealed envelopes) ensures high methodological rigor and minimizes selection bias, providing a high level of evidence for physical rehabilitation in hernia care. This study is, to our knowledge, the first to utilize the Pain Disability Index (PDI) specifically for inguinal hernia. This allows for a multidimensional assessment of how hernia symptoms affects life domains (social, occupational, and personal) beyond simple pain scores. By incorporating the Minimal Important Change (MIC) and the Youden Index, the study moves beyond mere statistical significance (*p* < 0.05) to define “responders”—patients who experienced a recovery that was personally meaningful. Supplementing subjective patient reports with objective clinical tests (Trunk Raising and Double Leg Lowering) provides a robust, measurable verification of abdominal wall recovery that is rarely documented in hernia literature. The implementation of a protocol starting four weeks preoperatively demonstrates that physical optimization *before* surgical trauma is as critical as postoperative recovery, effectively preparing the musculature for the procedure. Demonstrating a significant reduction in return-to-work time (9.28 vs. 12.86 days) provides a powerful economic argument for healthcare systems to adopt active recovery protocols to reduce work absenteeism. A mean follow-up of 18.4 months confirms that early, unrestricted activity and structured exercise do not increase recurrence rates or surgical complications, debunking traditional myths regarding long-term physical restriction. Finally, the identification of independent predictors (preoperative PDI, defect size, and symptom onset) via multivariate regression allows clinicians to identify and prioritize high-risk patients who would benefit most from rehabilitation.

### Limitations

Even if our study is a prospective randomised one it possess several inherent limitations related to logistical, scientific and ethical factors. The number of patients is not a large cohort although the Minimum Number to Treat (MNT) for statistical significance was correctly calculated and applied. Although randomization balances known and unknown confounders, small sample sizes can still result in imbalances between groups. Large cohorts prospectively randomised are necessary. An inclusion bias can be encountered because participants in the study may behave differently or be healthier than the general population, limiting the applicability of results. We limited our follow-up period only for the goal of the study (return to work) but long-term studies are prone to losing participants (dropping out, moving away, or death), which can lead to missing data and introduce selection bias (attrition bias); an extensive period is beneficial because recurrence and chronic groin pain can be clinically manifest even after 3 years. A selection bias was recorded because only one referral surgeon was involved in the study; this can be eliminated only by enrolling a population, which is almost a technical and financial impossibility. A potential bias can result from our inability to fully blind the participants even if a surgeon not involved in the study performed the postoperative assessment. In our evaluation, PDI was not associated with a psychical domain. Depression is an important factor and its presence is associated with an additional 9.4 days absence from work. Finally, probably not all patients were classified correctly to MIC_ROC_ because they have their own individual threshold.

## Conclusions

Symptomatic inguinal hernia is associated with a significant degree of disability and impaired abdominal wall functionality, which can be accurately assessed using the Pain Disability Index (PDI) and validated clinical tests (Trunk Raising and Double Leg Lowering). The integration of a structured physical rehabilitation program into the perioperative management of patients undergoing Lichtenstein tension-free repair significantly enhances functional recovery and clinical outcomes. Patients who followed a preoperative and postoperative exercise protocol returned to work significantly earlier (9.28 days) compared to the control group (12.86 days, *p* < 0.001). This combined approach facilitates accelerated postoperative recovery, shortened convalescence, and a significantly earlier return to work, even for patients engaged in heavy manual labor. Along with psychosocial factors, preoperative PDI scores serve as a critical predictive tool for determining whether a patient will achieve a rapid professional reintegration or remain on long-term disability. Our findings underscore the importance of shifting toward Patient-Reported Outcome Measures (PROMs) to evaluate surgical success. However, further large-scale studies are required to deepen our understanding of the effectiveness of physical rehabilitation on convalescence and to establish high-quality Minimal Important Change (MIC) thresholds across diverse patient populations.

## Supplementary material

Below is the link to the electronic supplementary material.

Supplementary File 1 (DOCX 17.1 KB)
