# Supplementary material for: The efectiveness of perioperative abdominal wall exercises upon functional recovery and return to work after Lichtenstein tension – free repair: a prospective randomized case –control study
Source: Hernia. 2026 Jun 2;30(1):239. doi: 10.1007/s10029-026-03729-0 (PMC13230312; doi:10.1007/s10029-026-03729-0)
Supplement: Supplementary file 1 — (DOCX 17.1 KB) [file 10029_2026_3729_MOESM1_ESM.docx]

1. disability

The preoperative level of disability for the patients with rehabilitation was higher than for the patients without and the difference was significant (56.7±2.51 *versus* 53.05±2.74; 95% CI of difference 2.82 – 4.47; *p*<0.001) (table 3). According to the degree of severity, in the study group 83 patients reported very severe states with values of the PDI between 52 and 69, while in the control group were reported 85 with PDI values between 51 and 59; no statistically significant association between the variables (χ^2^ = 0.18, *p*=0.673, Cramer’s V effect size 0.03). In the study group were 13 patients who reported extremely severe disability (PDI between 60 and 63). No valid correlation was found between the value of PDI and analysed independent variables (age, gender, BMI, comorbidities score index, trunk raising or double leg lowering). When the variable “heavy work” was forced in the model, a mild correlation was obtained but with good sensitivity and specificity (AUROC = 0.52; p=0.047, asymptotic 95%CI =0.402 – 0.637).

At the first postoperative evaluation (day 7), PDI decreased for both groups of patients and the decreased value was significant comparing to preoperative values (Table 3). The mean decreased value was higher for the patients from the study group (38.3±4.02) comparing to controls (39.47±3.25) and the difference was significant (p=0.004). There were more patients with a moderate grade of disability (PDI between 31 and 40 points) in the study group (70 patients) comparing to 62 patients in the control group but the difference was without significance ( χ^2^=1.52, p=0.218, Cramer’s V effect size =0.09). The mean value of the difference between preoperative and 7 days value of the PDI was 18.48 ± 4.31 (8 – 31) for the study group and 13.59 ± 3.83 for the control group (p<0.001). In the study group, Jouden index (J_max_) of the Minimal Important Change was 0.828 and positive if greater than 13 points (AUROC = 0.999; asymptotic significance p<0.001). Minimal Important Change for the control group was Jmax= 0.724 and positive if greater than 17 points (AUROC = 0.872; p<0.001). In the study group, 61 patients achieved MIC, while in the control only 42 (χ^2^= 7.471, p<0.006, Cramer’s V effect size = 0.27).

Final early evaluation of the PDI (30 days evaluation) revealed a slight decrease for both groups without any statistical significance (t-test p = 0.082); mean PDI for the study group was 33.08±4.97 and for the control 34.14±4.32. For both groups a mean decrease of 5 points was noted compared with the 7-day evaluation. If, at the 7-day evaluation no patients with minor degrees of disability were registered, at 30 days the number of those with moderate disability (21-30 points) reached 28 (29%) for the rehabilitation group and 14 (14%) for the control group; the chi-square test was statistically significant (χ² = 5.96, p = 0.015). At the same time, the number of patients with moderately severe disability (31 – 40 points) was lower in the study group (59 – 61%) compared to the control group (78 – 80%).

Except heavy work, no independent variable influenced the values of PDI in univariate analysis. In the logistic regression, heavy work did not influenced PDI values for none of the groups.

**Pain**

Minimal hernia pain was reported preoperatively during baseline testing, without differences between groups both at rest and during exercises. Mean value of pain during exercise was 1.9 ±0.1 (0 -2) respectively 2.1±0.1 (0-3) (p = 0.937).

For both parameters at rest and at mobilization, pain mean value on VAS was higher for controls at 24 hours after surgery (table 4). At 24 hours, severe pain (6 – 7 points VAS) was recorded for 7 patients in study group respective 16 patients for the controls (χ² = 4.00, p =0.046). At mobilization only 20 patients from study group complain for severe pain compare with 59 (χ² = 32.48, p < 0.001).

Value of pain at mobilization for both groups was correlated in univariate analysis with preoperative PDI, age, BMI, onset, defects larger than 3 cm and total score of abdominal wall functionality. When multivariate regression was applied the only confounders independently associated with the postoperative pain were PDI (OR = 0.0781, 95%CI = 0.034 – 0.62; p = 0.038), onset (OR – 0.074; 95%CI = 0.08 – 0.85; p = 0.027) and defects larger than 3 cm (OR – 0.059; 95%CI = 0.092 – 14.3; p = 0.003). All these confounders were associated with high specificity and sensitivity on ROC curves (data not shown).

**Abdominal wall functionality**

Abdominal wall functionality expressed as the sum of TR and DLL was poor for 29 patients (14.94%), fair for 96 patients (49.48%), good in 59 (30.41%) and normal only in 5 (2.57%). For five patients the function was trace. The distribution among study groups is represented in Table 5. For both group of patients, in univariate analyses, total score of the abdominal function (TS) was correlated with age, BMI, preoperative PDI and the onset of symptoms. In multivariate regression, independent confounders associated with TS were preoperative PDI (OR = 0.03; 95%CI = 0.021 – 0.458; p=0.021) and the onset of symptoms (OR = 0.0725; 95%CI = 0.032 – 0.69; p = 0.034).

The 7-day evaluation of the abdominal wall functionality revealed a small but insignificant increase comparing to preoperative value of the TS in the study group (5.34±1.29 vs 5.15 ±1.4 t-Student test p = 0.337). In the control group the mean value of the 7 day TS drastically decreased to 5.31±0.99 (t-Student test p<0.001). The mean values between the groups were not different statistically (t-Student test p=0.898). The distribution of the patients according to the degree of functional alteration was similar for both groups but in the control group an increased number with fair function was recorded compared to the preoperative score ( 64 versus 44; χ^2^ = 8.35; p = 0.003). This difference was not noted for the study group. For both groups of patients, TS was highly correlate in univariate regression with the preoperative PDI and with mean value of pain intensity at moving at 72 hours (r = 0.761; p =0.01 respective r = 0.662; p = 0.05); the association was highly sensitive and specific on ROC curve (AUROC = 0.792; p = 0.02 respective AUROC = 0.779; p = 0.03). In multilogistic regression the only independent associated confounder was preoperative PDI (OR = 0.03; 95%CI = 0.005 – 0.29; p = 0.0012).

At the end of the first month, abdominal wall functionality increased significantly compared to previous values for both groups, but the mean TS was higher for the study group compared with the mean value of the control group (7.77±1.02 versus 7.29±0.92; p<0.001). In the control group there was a significantly higher proportion of patients with fair function (5 – 6 points) compared to the patients of the study group (29 controls respective 10 study; χ^2^ = 11.58; p<0.001). Normal abdominal wall functionality was achieved by 22 patients from the study group compared to nine from the controls (χ^2^ = 6.48; p = 0.01). Patients with good function were equally distributed (65 versus 59; χ^2^ = 0.80; p = 0.368).

**Return to work**

The mean period for return to normal activities and work was 14.7±6.3 days (3 -21). In the rehabilitation group the mean period was 9.28±4.47 (3 – 21) days while for the control group was 12.86±5.16 days (5 – 21) and the difference was statistically significant (p<0.001). In the study group, 48 patients (49%) return to normal activity before 7 days compared to 21(22%) for the control group. The chi-square test of independence revealed a significant result, χ² = 16.40, p < 0.001, Cramer’s V = 0.29). In table six is detailed the distribution of patients according to the type of work.

Return to work was highly correlated in univariate analyse with the onset of symptoms (r =0.681; p = 0.02), preoperative PDI (r = 0.563; p =0.04), preoperative total score of functionality (r = 0.711; p = 0.01), BMI (r = 0.587; p=0.05) and age (r = 0.519; p = 0.05). Multivariate logistic regression return preoperative PDI (OR = 0.03; 95%CI = 0.01 – 0.74; p = 0.002), total score (OR = 0.004; 95%CI = 0.03 – 0.145; p<0.001) and onset of symptoms (OR = 0.041; 95%CI = 0.006 – 0.3; p =0.001) as independent variables influencing early return to work. The stepwise model was also statistically significant (P =0 .0001) and explained 61% of the variance. The previous predictors were forced into the model. An increase of the PDI with 10 points over MIC was associated with two additional day for heavy workers (p = 0.009) but with no extra days for light workers and retired (p = 0.02) in the study group and with four additional days for the controls (p=0.005). A two points decrease of the total score of functionality was also associated with two additional days for heavy workers (p = 0.0027) in the rehabilitation group and with 5 days in the control (p = 0.004). The onset of symptoms showed no association.

**Follow-up and outcomes**

All patients were examined at the above-mentioned interval of time. The mean follow up for all patients was 18.4±3.6 months. At 12 months, the operative surgeon clinically examined all patients. During the follow-up period next complications were encountered: hematoma – 3 patients; seroma – 9 patients, wound dehiscence – 1 patient and chronic pain – 2 patients. The complications were minor and they did not need surgical intervention. There was no difference between the groups in terms of local events. No recurrence was clinically diagnosed within the follow-up period. During physical rehabilitation, no acute accident of the hernia was encountered.
